# Neutrophil-lymphocyte ratio as prognostic factor in metastatic breast cancer

**DOI:** 10.61622/rbgo/2026rbgo10

**Published:** 2026-05-12

**Authors:** Gustavo Motta Cabello dos Santos, Nereida Kilza Costa-Lima, Isabela da Mata Tiezzi, Giovana Kobayashi Pavão, Israel Geraldo Silva, Priscila Malara Maia, Hélio Humberto Angotti Carrara, Daniel Guimarães Tiezzi

**Affiliations:** 1 Universidade de São Paulo Faculdade de Medicina de Ribeirão Preto Departamento de Ginecologia e Obstetrícia Ribeirão Preto SP Brazil Departamento de Ginecologia e Obstetrícia, Faculdade de Medicina de Ribeirão Preto, Universidade de São Paulo, Ribeirão Preto, SP, Brazil.

**Keywords:** Neutrophil-to-lymphocyte ratio, Metastatic breast cancer, Prognostic biomarker, Systemic inflammation, Cancer survival

## Abstract

**Objective:**

The aim of the study was to analyze the role of the neutrophil to lymphocyte ratio (NLR) as a prognostic factor in metastatic breast cancer (MBC).

**Methods:**

A retrospective cohort study including 359 recurrent MBC patients was performed. Clinical and pathological attributes were retrieved from patients’ medical files and their importance in the overall survival were evaluated using the Cox univariate and multivariate proportional hazard models. A confidence interval of 95% was used to select significant variables and the Wald test was used to infer the best NLR cutoff value to discriminate into low and high categories.

**Results:**

In univariate analyses, the variables estrogen receptor (ER) and progesterone receptor (PR) expressions, previous treatment with adjuvant chemo, NLR, PLR (platelet-lymphocyte ratio), age at diagnosis and the main metastatic site (bone/lymph node, visceral or central nervous system) variables were significant to determine the hazards. After filtering the collinear variables, the final model was tested with 6 variables based on 339 patients and the NLR at the time of the recurrence diagnosis (NLR_rec), the hormone receptor (ER or PR) status and the age at diagnosis were considered significant. The best NLR_rec value to discriminate against patients was 4.43 (Wald test= 51.7) and there were 239 patients categorized as low and 111 as high NLR category. The median overall survival time was 18 and 4 months in NLR-low and NLR-high groups, respectively (p< 0.001).

**Conclusion:**

NLR is an independent prognostic factor in recurrent MBC patients.

## Introduction

Breast cancer is among the most common malignancies affecting women worldwide, with metastatic breast cancer (MBC) significantly contributing to cancer-related mortality.^(1)^ MBC, characterized by the spread of cancer to distant organs, poses substantial treatment challenges even amidst advancements in targeted therapies and immunotherapy. Identifying prognostic factors for MBC is critical for guiding clinical decisions, enabling personalized treatment, and potentially improving patient outcomes.

Traditionally, prognostic markers such as hormone receptor (HR) status, HER2 expression, and tumor grade have been used to assess breast cancer risk. HR-positive and HER2-negative subtypes generally progress more slowly, whereas triple-negative and HER2-positive tumors exhibit more aggressive behaviors.^(2)^ Additional factors, including tumor size, lymph node involvement, and markers like Ki-67, provide insights but often do not fully capture the complexities of immune responses and systemic inflammation associated with metastasis.

Recent research has highlighted the potential of systemic inflammation markers, especially the neutrophil-lymphocyte ratio (NLR), as prognostic tools across multiple cancers.^(3)^ NLR, calculated from the absolute neutrophil and lymphocyte counts, reflects the balance between pro-tumor inflammation and anti-tumor immune responses. Elevated NLR is linked to poorer outcomes in cancers such as colorectal, lung, gastric, and breast cancer.^(4)^ This association is likely due to neutrophils’ role in supporting tumor growth and metastasis, contrasting with lymphocytes’ critical role in tumor surveillance and immune defense.^(5)^

In breast cancer, high NLR is particularly associated with reduced survival in patients with advanced or metastatic disease.^(6)^ NLR offers a cost-effective, accessible marker that reflects systemic immune responses, potentially complementing molecular markers in assessing disease progression. However, existing studies largely focus on single time-point NLR measurements. By examining NLR both at diagnosis and after metastasis, particularly in chemotherapy-treated patients, this study aims to provide dynamic insights into the prognostic value of NLR. Our findings could contribute to establishing NLR as a valuable, accessible prognostic tool for managing MBC alongside current molecular and clinical predictors.

## Methods

This is a retrospective observational study including 359 recurrent MBC patients from Hospital das Clínicas - Ribeirão Preto School of Medicine diagnosed between 2012 and 2024.

Clinical and pathological data were collected from patient medical records, focusing on variables considered relevant to prognosis. Key data included patient demographics, such as age at diagnosis, as well as clinical details like date of diagnosis, date of relapse, last follow-up date, and survival status. Hormone receptor status was recorded through estrogen and progesterone receptor levels, including their respective expression percentages, along with HER2 status, Ki-67 index, and tumor grade. Staging information covered tumor (T), nodal (N), and metastasis (M) classifications, and treatment history included records of chemotherapy and surgical interventions. The main site of metastasis was documented to account for differences in metastatic patterns. Inflammatory markers were a key focus, particularly absolute neutrophil and lymphocyte counts, recorded both at initial diagnosis and after recurrence, along with platelet counts. The neutrophil-to-lymphocyte and platelet-to-lymphocyte ratios were estimated at the time of the diagnosis (NLR and PLR, respectively) and at the time of recurrence diagnosis (NLR_rec and PLR_rec, respectively).

Overall survival was the primary outcome, analyzed in association with these attributes using Cox proportional hazards models. Univariate analyses identified significant variables, which were then included in a multivariate model to control for confounding effects and evaluate independent predictors of survival. Collinear variables were excluded from the final model to maintain robustness. A 95% confidence interval was applied to assess significance, and the Wald test determined the optimal cutoff for the NLR_rec, enabling categorization into low and high NLR groups to stratify patient risk. Statistical significance was set at a p-value of < 0.05, and all analyses were performed in R software.^(7)^

The study was based on the information retrieved from patients’ files and was approved by the local committee in ethics (CAAE: 84282224.8.0000.5440, number 7.208.932).

## Results

The median overall survival time was 12.3 months. In univariate analyses, the variables ER expression, PR expression, NLR, NLR_rec, PLR_rec, age at diagnosis and the main metastatic site (bone/lymph node, visceral or central nervous system) variables were significant to determine the hazards (Table 1).

**Table 1:** Demographic, clinical and laboratory parameters related to prognosis in metastatic breast cancer - Univariate Cox Regression model

Characteristic		n	HR	95% CI	p-value
Age		359	1.01	1.00, 1.02	0.004
Race		359			
	B		—	—	
	P		1.20	0.92, 1.57	0.2
Stage		359			
	1		—	—	
	2		1.08	0.64, 1.85	0.8
	3		1.32	0.80, 2.19	0.3
Grade		336			
	1/2		—	—	
	3		1.28	1.00, 1.64	0.051
ER		357			
	Negative		—	—	
	Positive		0.58	0.46, 0.72	<0.001
PR		357			
	Negative		—	—	
	Positive		0.68	0.54, 0.84	<0.001
HER2		355			
	Negative		—	—	
	Positive		1.00	0.78, 1.27	>0.9
Chemo		359			
	no		—	—	
	yes		0.78	0.58, 1..04	0.092
NLR		354	1.08	1.02, 1.14	0.006
PLR		354	1.00	1.00, 1.00	0.2
NLR_rec		350	1.07	1.05, 1.08	<0.001
PLR_rec		354	1.00	1.00, 1.00	<0.001
Main site		349			
	bone/lymph node		—	—	
	cns/visceral		1.44	1.15, 1.79	<0.001
Nº Distant Sites		341			
	1		—	—	
	>1		0.94	0.74, 1.20	0.6
Relapse TIme		359	1.00	1.00, 1.00	0.2

Abbreviations: CI = Confidence Interval, HR = Hazard Ratio, ER= Estrogen Receptor, PR= Progesterone Receptor, NLR= Neutrophil-Lymphocyte ratio, cnc= central nervous system, PLR= Platelet-Lymphocyte ratio, CHEMO= use of neoadjuvant or adjuvant chemotherapy

After filtering the collinear variables, the final model was tested with 6 variables based on 339 patients and the NLR, the hormone receptor (ER or PR) status and the age at diagnosis were considered significant (Table 2).

**Table 2:** Demographic, clinical and laboratory parameters related to prognosis in metastatic breast cancer - Multivariate Cox regression model

Characteristic		HR^2^	95% CI^2^	p-value
Hormone receptor				
	Positive	ref	—	
	Negative	1.41	1.10, 1.81	0.007
NLR		1.05	0.99, 1.12	0.10
NLR_rec		1.06	1.04, 1.08	<0.001
Main site				
	bone / lymph node	ref	—	
	cns / visceral	1.34	1.06, 1.69	0.015
Age		1.01	1.00, 1.02	0.001
PLR_rec		1.00	1.00, 1.00	0.15

1 HR = Hazard Ratio, CI = Confidence Interval, NLR= Neutrophil-Lymphocyte ratio at primary diagnosis, NLR_rec= Neutrophil-Lymphocyte ratio at recurrence, cnc= central nervous system, PLR_rec= Platelet-Lymphocyte ratio at recurrence

The best NLR_rec value to discriminate against patients was 4.43 (Wald test= 51.7) and there were 239 patients categorized as LOW and 111 as HIGH NLR category. Patients with high NLR were more likely to present with higher tumor grade (Grade 3) compared to those with low NLR (39% vs. 24%, p = 0.008). Hormone receptor status also demonstrated significant differences. High NLR were associated with progesterone and estrogen receptor expression. The prevalence of ER and PR negative in NLH high and low patients were 45% versus 31% (p = 0.008) and 58% versus 43% (p= 0.009), respectively. Additionally, the distribution of metastatic sites differed significantly between the two groups. Patients with high NLR had a higher frequency of central nervous system and visceral metastases compared to bone / lymph node dissemination (62% vs. 44%, p = 0.002). No significant differences were observed regarding age, race, tumor stage, HER2 status, prior chemotherapy exposure, number of metastatic sites, or time to relapse (Table 3).

**Table 3:** Clinical and pathological characteristics according to the neutrophil-to-lymphocyte ratio (NLR) estimated at the diagnosis of the metastatic recurrence

Characteristic		N	NLR high N = 111^1^ (%)	NLR low N = 239^1^ (%)	p-value^2^
Age		350	54.5 (22.2)	52.6 (18.5)	0.085
Race		350			0.13
	White		95 (86)	188 (79)	
	Black		16 (14)	51 (21)	
Stage		350			0.9
	1		7 (6.3)	13 (5.4)	
	2		27 (24)	63 (26)	
	3		77 (69)	163 (68)	
Grade		327			0.008
	1/2		62 (61)	171 (76)	
	3		39 (39)	55 (24)	
ER		348			0.008
	negative		51 (46)	74 (31)	
	positive		60 (54)	163 (69)	
PR		348			0.009
	negative		64 (58)	101 (43)	
	positive		47 (42)	136 (57)	
HER2		346			0.8
	negative		80 (73)	168 (71)	
	positive		30 (27)	68 (29)	
Chemo		350			0.5
	no		21 (19)	38 (16)	
	yes		90 (81)	201 (84)	
Main Site		341			0.002
	bone/lymph node		41 (38)	132 (56)	
	cns/visceral		66 (62)	102 (44)	
Nº Dist SIte		333			0.5
	1		36 (34)	67 (30)	
	>1		71 (66)	159 (70)	
Relapse time (days)		350	775.0 (896.5)	809.0 (1,186.0)	0.7

1 Median (IQR); n (%)

2 Wilcoxon rank sum test; Pearson's Chi-squared test; Fisher's exact test ER= Estrogen Receptor, PR= Progesterone Receptor, NLR= Neutrophil-Lymphocyte ratio at the time of the recurrence diagnosis, cnc= central nervous system, Chemo= use of neoadjuvant or adjuvant chemotherapy

**Figure 1:**
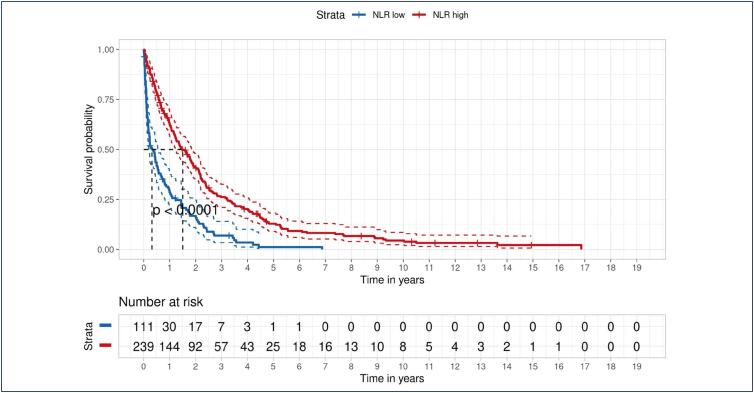
Kaplan-Meier survival curves stratified by neutrophil-to-lymphocyte ratio (NLR) in patients with recurrent metastatic breast cancer

We analyzed overall survival comparing patients with low and high NLR. The median overall survival time was 18 and 4 months in NLR-low and NLR-high groups, respectively (p< 0.0001) (Figure 1).

## Discussion

In this study, we demonstrated that a high neutrophil-to-lymphocyte ratio (NLR_rec) was shown to be a possible independent prognostic factor for reduced overall survival in patients with recurrent metastatic breast cancer (MBC). Our results reinforce and expand upon previous findings that systemic inflammatory markers, particularly NLR, hold significant prognostic value across various malignancies, including breast cancer.

Elevated NLR reflects a pro-tumor systemic inflammatory state characterized by neutrophilia and relative lymphopenia. Neutrophils are known to support tumor progression through the secretion of growth factors, proteases, and proangiogenic cytokines, while also suppressing the anti-tumor immune response mediated by lymphocytes.^(8)^ Conversely, lymphocytes are critical players in tumor immunosurveillance, and their reduction has been associated with impaired antitumor immunity and unfavorable prognosis.^(9)^ These mechanisms are well supported in the literature and provide a biological rationale for the observed association between high NLR and poor survival outcomes in cancer patients.^(10)^

Our data align with prior studies that observed a significant correlation between elevated NLR and worse survival in MBC. For instance, Ethier et al. (2017)^(11)^ reported that patients with high NLR had significantly shorter progression-free and overall survival, independently of classical prognostic markers, supporting the integration of NLR into routine clinical assessments. Similarly, Koh et al. (2015)^(12)^ found that NLR was an independent predictor of poor prognosis in HER2-positive MBC patients treated with trastuzumab, indicating the broader applicability of NLR beyond hormone receptor status.

In our cohort, we identified an optimal NLR cutoff of 4.43, above which patients had a markedly reduced median overall survival of 4 months compared to 18 months in the low NLR group (p<0.001). This cutoff is consistent with those reported in previous literature, which commonly range between 3 and 5.^(13,14)^ The high statistical significance of the NLR stratification further reinforces the robustness of NLR as a discriminative prognostic biomarker in this setting.

In addition, our multivariate analysis confirmed that NLR remained an independent prognostic factor after controlling for hormone receptor status, age at diagnosis and the main site of metastasis. These results highlight that systemic inflammation, as measured by NLR, provides complementary prognostic information to traditional clinicopathological factors. Notably, the prognostic impact of NLR was independent of whether the patients received adjuvant or neoadjuvant chemotherapy, which suggests that NLR may retain its predictive value in different treatment contexts.

Our findings also align with the growing recognition of systemic inflammation as a key contributor to breast cancer progression and immune evasion, as extensively reviewed by Templeton et al. (2014)^(3)^ and Zahorec (2001),^(8)^ who both advocate for the integration of inflammatory markers into cancer prognostication frameworks. Despite this, the clinical application of NLR is still limited, and there is a need for standardized cutoff values and prospective validation in diverse patient populations.^(14,15)^

A potential limitation of our study is its retrospective design and the reliance on data from a single institution, which may limit the generalizability of our findings. Additionally, the dynamic evolution of NLR during the disease course and its modulation by treatment were not fully addressed, warranting further investigation. Nevertheless, our study provides additional evidence supporting the utility of NLR as an accessible, cost-effective prognostic tool in MBC.

## Conclusion

Our study helps to analyze the possible use of NLR_rec as an independent and readily available prognostic marker in patients with recurrent MBC. Due to its low cost and ease of assessment, NLR could be integrated into clinical practice to improve risk stratification and guide treatment and palliative care decisions. Future prospective studies should aim to validate these findings and explore the role of NLR as a possible biomarker to monitor systemic inflammation and response to therapy in patients with MBC.

## Data Availability

The research data are described in the article presented.
